# Technological prospecting of patents related to monitoring accidents due to falls in hospitals

**DOI:** 10.1590/0034-7167-2023-0084

**Published:** 2024-02-26

**Authors:** Renata Camargo Alves, Rosana Maria Barreto Colichi, Silvana Andrea Molina Lima

**Affiliations:** I Universidade Estadual Paulista Júlio de Mesquita Filho. Botucatu, São Paulo, Brazil

**Keywords:** Accidental Falls, Technology, Patent, Hospitals, Inventions, Accidentes por Caídas, Tecnología, Patente, Hospitales, Invenciones, Acidentes por Quedas, Tecnologia, Patente, Hospitais, Invenções

## Abstract

**Objectives:**

to map the production of technologies aimed at monitoring falls in a hospital environment protected by registered patents.

**Methods:**

a technological prospecting of international patents, with a quantitative approach, with search carried out between February and March 2022 in the Derwent Innovations Index database with descriptors fall, hospital, monitoring.

**Results:**

212 patents were found, with the majority filed and published since 2010, by Tran B (9) and Cerner Innovation Inc (9), focused on health technology. Universities were responsible for 13% of deposits. There was a predominance of records from the United States (43.4%), China (21.7%) and Japan (12.3%), in addition to technological strategies classified as devices for the environment (80.7%) and for preventing falls (66.5%) as well as trend towards resources with multiple functionalities in the same technology.

**Conclusions:**

the plurality of functions in the same device reflects the search for optimizing resources and the concern with comprehensive care.

## RESUMO

**Objetivos:**

mapear a produção de tecnologias voltadas para monitoramento de quedas em ambiente hospitalar protegidas por patentes registradas.

**Métodos:**

prospecção tecnológica de patentes internacionais, com abordagem quantitativa, com busca realizada entre fevereiro e março de 2022 na base *Derwent Innovations Index* com os descritores *fall, hospital, monitoring*.

**Resultados:**

foram encontradas 212 patentes, com maioria depositada e publicada a partir de 2010, por Tran B (9) e *Cerner Innovation Inc* (9), focadas em tecnologia em saúde. As universidades foram responsáveis por 13% dos depósitos. Houve predomínio de registros dos Estados Unidos (43,4%), da China (21,7%) e Japão (12,3%), além de estratégias tecnológicas classificadas como dispositivos para ambiente (80,7%) e para prevenção de quedas (66,5%), bem como a tendência de recursos com múltiplas funcionalidades em uma mesma tecnologia.

**Conclusões:**

a pluralidade de funções em um mesmo dispositivo reflete a busca pela otimização dos recursos e a preocupação com a integralidade do cuidado.

## RESUMEN

**Objetivos:**

mapear la producción de tecnologías destinadas al monitoreo de caídas en un ambiente hospitalario protegido por patentes registradas.

**Métodos:**

prospección tecnológica de patentes internacionales, con enfoque cuantitativo, con búsqueda realizada entre febrero y marzo de 2022 en la base de datos *Derwent Innovations Index* con los descriptores caída, hospital, monitoreo.

**Resultados:**

se encontraron 212 patentes, la mayoría presentadas y publicadas desde 2010, por Tran B (9) y *Cerner Innovation Inc* (9), enfocadas en tecnología de la salud. Las universidades fueron responsables del 13% de los depósitos. Hubo predominio de registros de Estados Unidos (43,4%), China (21,7%) y Japón (12,3%), además de estrategias tecnológicas catalogadas como dispositivos ambientales (80,7%) y para prevenir caídas (66,5%), así como la tendencia hacia recursos con múltiples funcionalidades en una misma tecnología.

**Conclusiones:**

la pluralidad de funciones en un mismo dispositivo refleja la búsqueda de optimización de recursos y la preocupación por la atención integral.

## INTRODUCTION

Providing unsafe care can have consequences for patients as well as financial implications for the health system and consequently for a country’s economy^(1)^. Therefore, the global concern with reducing health care costs and the search for results and indicators that demonstrate the quality and safety of patient care are topics in evidence on the global stage^(2)^. Complexity of care, punitive cultures, unpreparedness of professionals and lack of standards in carrying out procedures are factors that make hospital institutions vulnerable to the occurrence of errors^(1)^.

Among adverse events, fall accidents cause considerable impacts on morbidity and mortality rates, in addition to representing an economic burden for society and hospital institutions^(3)^. They represent the second leading cause of deaths from unintentional injuries, with adults over 60 years of age being the population that suffers the highest number of fatal falls^(4)^. In Brazil, the occurrence of this adverse event was also responsible for the increase in hospital admission, mortality and lethality rates in older adults^(5)^. Although its occurrence is directly proportional to increasing age, injuries resulting from this event cannot be considered an inevitable part of aging^(6-7)^.

Hospital admission is considered one of the factors that results in an increased risk of falling, due to a change in environment and unfamiliarity with the location. In this regard, the length of stay in the hospital proportionally affects this event, i.e., the longer the hospital stay, the greater the risk of falling^(8)^.

Thus, research to understand causes, consequences and amounts spent on these events provides support for establishing improvement plans, implementing interventions and developing policies in health organizations^(4-5,8-9)^.

In these environments, prevention strategies that can be adopted include patient and family educational processes, care team training and fall risk assessment. However, such conduct must be part of planning and broad patient safety policies, creating safer environments and also including investment in technological resources with the aim of mitigating this event^(4,10)^. In periods of cost containment, health services should not invest resources in costly innovations without evidence showing the intervention effectiveness and the return on this investment^(9)^.

Among technological means, we have the expansion of the use of fall detection resources, with the prevalence of products aimed at signaling the moment of the incident, with the aim of providing assistance in the shortest possible time. However, there is a change in focus towards fall prevention^(7)^. Internationally, there is an expansion in the use of monitoring resources which, despite being recent, demonstrate excellent results in care practice, reducing the incidence of falls, in addition to increasing family and care team satisfaction. However, the number of nursing studies focused on this type of technology is still discreet, requiring further research on the subject^(9,11-12)^.

Knowing and analyzing innovative solutions on the market by studying patents can help disseminate resources aimed at monitoring and preventing patient falls^(12)^.

## OBJECTIVES

To map the production of technologies aimed at monitoring falls in a hospital environment protected by registered patents.

## METHODS

### Ethical aspects

The study was approved by the Research Ethics Committee of the Faculty of Medicine of Botucatu of the *Universidade Estadual Paulista Júlio de Mesquita Filho* (UNESP). The Informed Consent Form was not applied, as the study did not involve human beings.

### Study design, period and place

This is a technological patent prospecting research with a quantitative approach, whose sample was made up of international patent registrations relating to fall monitoring in the hospital environment.

The search was carried out between February and March 2022, with a sample space from 01/01/2000 to 02/08/2022, in the Derwent Innovations Index (DII) database, available from the Web of Science interface with access via Coordination for the Improvement of Higher Education Personnel (CAPES - *Coordenação de Aperfeiçoamento de Pessoal de Nível Superior*)^(13)^.

### Sample; inclusion and exclusion criteria

The study included all patent registrations related to preventing and signaling falls to monitor patients in a hospital environment, published between 2000 and 2022, a time frame that culminates with the publication of “To Err is Human”^(9)^, a document that awakened and revolutionized global concern about “patient safety” and treatments related to adverse events. Registrations intended exclusively for the domestic environment and devices that were not intended for patient monitoring were excluded.

### Study protocol

Technological prospecting studies consist of mapping technologies, highlighting their current state and how they fit into society, also providing support for decision-making through the analysis of their information. Furthermore, this survey serves as a basis for understanding the market and obtaining guiding data for scientific research, highlighting relevance and gaps that can be filled by new inventions and preventing existing products from being replicated without clear improvements^(14-16)^.

A patent corresponds to a temporary right to creation, granted to an inventor or its holder, with territorial validity, requiring a description of technical and innovative content to be made available for public knowledge^(17)^.

Among the databases for searching patents, the Derwent World Patent Index (DWPI) is one of the most comprehensive databases, composed of records analyzed and indexed manually by world-renowned experts, offering resources that include a combination of exclusive information.

The methodological steps consisted of defining research strategies and descriptors, data collection, analysis of the information contained in the patent documents collected, selection of records relevant to the study objective, generation of a document with the results obtained, treatment and analysis of these data^(14)^.

The advanced search feature available on the patent consultation home page was used. In the “Add terms to query view” field, select the “Topic” option, which carries out the investigation using descriptors in the title, abstract, author keywords and Keywords Plus. The descriptors fall, hospital, monitoring were used together with the Boolean operator “AND”, a resource available on the query page, resulting in the search view ((TS=(fall)) AND TS=(hospital)) AND TS=(monitoring).

To obtain data from the records, the “Export” feature, “Tab-delimited file”, the “Records from 1 to 1000” option and “Save content” in the “Custom selection” option were used. On the “My Custom Export Selections (Derwent Innovations Index)” screen, the following items were selected: Inventor(s); Title; Patent number(s); Depositor(s); International Patent Classification (IPC); Class codes in Derwent; and Manual codes in Derwent. The selected data was exported in full into a Microsoft Office Excel^®^ table.

After reading the title, the complete summary and the documentation available on the database page, the researchers assessed whether the record was applicable to the study objective. For some documents, a virtual text translation service was used to analyze files that were not in English, Portuguese or Spanish. Information on patent filing date, date of international publication, language of publication of registration and patent nationality were obtained from these readings and included in the spreadsheet.

DII also provides information on the number of times a patent was cited by other registries. Considering that this quote is used to support the description and functionalities of the technological strategy, the ten most cited patents were listed in the description of other records.

### Data analysis, and statistics

Data were tabulated using Microsoft Office Excel^®^, with descriptive analysis carried out using graphs and tables, presenting the absolute/relative frequencies.

## RESULTS

**Figure 1:**
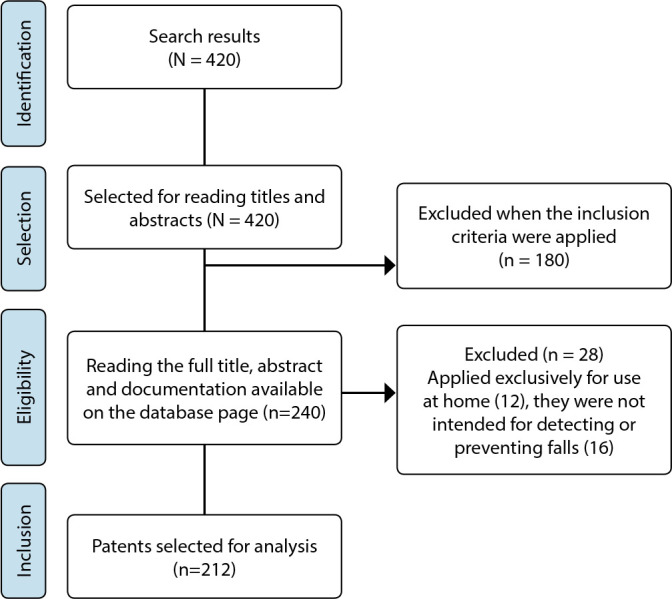
Flowchart of the selection process of patents available in the Derwent Innovations Index for sample composition, Botucatu, São Paulo, Brazil, 2022

The initial search resulted in 420 records, 208 of which were excluded after reading the title, the complete abstract and the abstract of documentation available on the database page. The final sample consisted of 212 patents, as shown in Figure 1.

**Figure 2:**
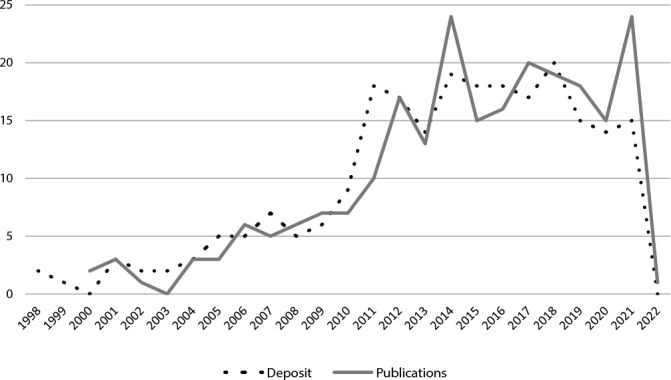
Evolution of patent filings and publications for fall prevention, Botucatu, São Paulo, Brazil, 2022

The majority of patents were filed and published in 2010, as can be seen in Figure 2, which shows the evolution of international patent publications for fall prevention.

Regarding inventors, Bao Q Tran, commercial and intellectual property lawyer, was responsible for the majority of inventions, with nine registrations (4%), also acting in the role of angel investor, a term currently used in the business world to designate investors in innovative companies with growth potential (startups). Al-Ali A was responsible for four registrations (2%), who is an engineer and works at Masimo Corporation, a company that develops monitoring technologies, responsible for filing all the patents of the aforementioned inventor.

Among patent filers, Bao Q Tran stands out, with nine deposits (4%), previously mentioned as the inventor with the highest number of patents, and the companies Cerner Innovation Inc, with nine (4%), Konink Philips NV, with seven (3%), and Hill-Rom Services Inc, with seven (3%), focused on health technology. Universities were responsible for 26 (13%) of deposits.

English was the most adopted language in the registrations, being present in 53% of them. However, oriental languages such as Chinese, Japanese and Korean prevailed in 41.5% of the sample.

There was a predominance of registrations coming from the United States (43.4%), followed by China (21.7%), Japan (12.3%) and Korea (8%). European countries also contributed to technological innovations in the field studied, being responsible for 8% of registrations, as shown in Table 1.

**Table 1:** Distribution of patent registrations by filing nationality, Botucatu, São Paulo, Brazil, 2022

Deposit nationality	n	%
United States	92	43.4
China	46	21.7
Japan	26	12.3
Korea	17	8.0
Australia	8	3.8
Germany	5	2.4
France	4	1.9
India	4	1.9
United Kingdom	2	0.9
Brazil	1	0.5
Belgium	1	0.5
Spain	1	0.5
Ireland	1	0.5
Luxembourg	1	0.5
Singapore	1	0.5
Sweden	1	0.5
Ukraine	1	0.5
Total	212	100

There was a predominance of technological strategies classified as environmental devices, representing 81% (171) of the sample, followed by wearable devices, with 19% (41). Regarding applicability, 66.5% (141) of patents were aimed at preventing falls, and 33.5% (71), for detecting the event.

The ten most cited patents in the description of other registrations were listed in Chart 1.

**Chart 1:** Distribution by patent number, registration name and number of registrations that cited the current patent registration, Botucatu, São Paulo, Brazil, 2022

Seq	Country	Patent title	Description	Type of technology	Nº of citations
D01	USA	Heart monitoring system for use by e.g. doctor, has wearable appliance with wireless transceiver to monitor patient movement, where heart disease recognizer transmits heart sound to remote listener if recognizer identifies problem	Cardiac monitoring system for medical use. It has a wearable device with a wireless transceiver to monitor patients’ movement and a heart disease recognition device that transmits the sound of the heart for the remote listener to identify the problem.	Wearable device	245
D02	USA	Monitoring device for detecting presence or absence of person in bed, has alarm that is actuated when characteristics such as relative position, velocity, acceleration exceeds preset threshold values	Monitoring device to detect the presence or absence of a person in the bed. It has an alarm that is triggered when characteristics such as relative position, speed and acceleration exceed predefined limit values.	Environmental monitoring device	164
D03	USA	Processing system for medical devices used for monitoring vital signs and motion for e.g. patient has processing component to determine alarm rule, determined by collectively processing first and second alarm conditions with alarm algorithm	Processing system for medical devices used to monitor vital signs and movement. It has a processing component to determine the alarm rule.	Wearable device	128
D04	USA	Healthcare monitoring system for e.g. hospital, has monitor with sensors to detect physiological data, location and status of wearer of monitor, with processing and storage capabilities for processing and storing data	Health monitoring system. It has a monitor with sensors to detect physiological data, location and user status, with data processing and storage capabilities.	Wearable device	114
D05	USA	Wireless monitoring system for bedridden patients in nursing home, has weight sensor pad to produce signal, when patient rises from bed, to activate alarm indicating patient room number in nurses station	Wireless monitoring system for bedridden patients. It has a weight sensor to produce a signal when patients get up from the bed, activating the alarm and indicating the patients’ room number at the nursing station.	Environmental monitoring device	102
D06	USA	Wireless physiological sensor for measuring acceleration of patient has first aperture of base and first through-hole via of substrate layer filled with at least thermally conductive material	The wireless physiological sensor to measure patient acceleration. It can be used as a wearable wireless sensor for pressure ulcer risk, fall detection or fall risk. The patient monitor sounds an alarm or alert to notify caregivers of the identified risk in an effort to anticipate and therefore prevent a patient fall when the patient monitor determines that patients’ fall risk is above a predetermined limit.	Wearable device	97
D07	USA	Wearable sensor i.e. temperature sensor, for use in health care monitoring system, has microcontroller coupled in communication with timing mechanism and sensor pairs, and accelerometer coupled to microcontroller	Wearable temperature sensor for use in health monitoring system. It has a microcontroller coupled in communication with a timing mechanism and pairs of sensors and an accelerometer coupled to the microcontroller.	Wearable device	84
D08	USA	Method for detecting when monitored individual has crossed outside of designated electronic perimeter, involves alerting remote computerized communication system when specific individual has crossed over designated perimeter	Method for detecting when monitored patients have crossed the designated electronic perimeter, issuing an alert to the remote computerized communication system when they cross the designated perimeter.	Environmental monitoring device	55
D09	USA	Method for detecting when monitored patient is crossed outside of designated perimeter, involves alerting computerized communication system when system detects that individual located in room is crossed over designated electronic perimeter	Method to detect when monitored patients cross a demarcated perimeter. It triggers an alert signal when the system detects that individuals have crossed the designated electronic perimeter.	Environmental monitoring device	41
D10	USA	Method for detecting need of medical assistance by monitored individual within e.g. hospital room, involves contacting individual or caregiver from computerized communication system in communication with computerized monitoring system	Method for detecting the need for assistance to a patient monitored in a hospital environment. It triggers a warning to the caregivers who will be connected to the computerized communication system.	Environmental monitoring device	39

By analyzing the devices classified as top 10, wearable technologies were found, such as watches or devices attached to the body (D01, D03, D04 and D07).

Some products had the function of checking vital signs, such as blood pressure, heart rate, respiratory rate, saturation and temperature, while being able to simultaneously analyze the state of activity, in addition to detecting and signaling the occurrence of a fall (D01 and D03).

It was also possible to learn about technologies that work by capturing images, which analyze their results and signal them in a monitoring center (D02 and D05) as well as equipment programmed to detect patients’ absence in bed or chair and signal the event (D05 and D06). Another instrument, in addition to detecting the risk and occurrence of a fall, also monitors the pressure in areas of contact between the body and the mattress, working to reduce the risk of developing pressure injuries(D06).

It was observed that some devices found in patent survey, in addition to verifying the risk and occurrence of falls, offer the functionality of reducing clinical deterioration, by simultaneously monitoring vital signs. Others provide body angulation as well as applicability for preventing the development of pneumonia, choking on food or medication. One of the pieces of equipment monitors vital signs and falls during evacuation, and can be applied to patients with a nursing diagnosis of constipation in which the force to evacuate can cause physiological changes and, possibly, a fall; another device automatically turns on the room light when in the standing position and turns it off when it detects that patients have laid down.

## DISCUSSION

The study revealed that the majority of patents were registered from 2010 onwards, in countries such as the United States, China, Japan and Korea, with technological strategies classified as devices for environments and aimed at preventing falls, although the most cited refer to wearable equipment. It is worth highlighting the plurality of functions present in the same device.

The publication of the “To Err is Human” (2000) report generated global notoriety on the subject of patient safety and concern about the creation of technological devices aimed at safe care^(18)^. However, the greatest growth occurred only ten years later. In Brazil, although the movements began in 2001 with the creation of Sentinel Hospitals, the Brazilian Nursing and Patient Safety Network (REBRAENSP - *Rede Brasileira de Enfermagem e Segurança do Paciente*) (2008) and the Brazilian National Patient Safety Program (PNSP - *Programa Nacional de Segurança do Paciente*) (2013), we still observed few products developed and registered^(19)^.

The predominance of patents coming from the United States reinforces data presented in the Global Innovation Index Report, which lists North America as the most innovative region in the world. The same document reinforces that, following the global economic decline resulting from the COVID-19 pandemic, a historic record in patent applications was observed in 2020, driven by countries such as China, Korea and the United States^(20)^.

The most prominent fields in these requests were in the areas of medical technology, pharmaceuticals and biotechnology, while sectors such as informatics, audiovisual technology and digital communication, which have always stood out, showed a significant drop. Everything indicates that the health sector took advantage of this moment to update the commercial potential of its inventions. Furthermore, the pandemic left its traces on the world stage, accelerating the geographic shift of innovation activities to Asia, even as continents such as North America and Europe still host some of the world’s leading innovators^(20)^.

In the current scenario of globalized economy, this study revealed the importance of the role of angel investors in the development of technologies aimed at health. Represented mainly by professionals who dedicate part of their resources and time to businesses that are in their initial phase, their work aims to share networks, knowledge and experience, adding value to the company. Can encourage innovative projects that are normally considered risky for the financial market in exchange for company shares^(21)^.

Study statistics showed a discreet participation of universities (13%) in record deposits, corroborating Brazilian research data that showed that, even with the advent of the professional master’s degree in nursing, we did not have an increase in the number of patent publications^(22)^. On the other hand, the increase in publications of journals focused on the entrepreneurial university approach, supported by a business culture, reveals a concern with the development of new technologies and the use of resources in an innovative way^(23)^.

Considering the technical and financial losses resulting from falls, there is a need for investment in technological development aimed not only at signaling and immediate assistance, but mainly at predicting and preventing falls, as this is a more promising perspective for avoiding injuries and its consequences. In this regard, we find a variety of resources available on the market developed for these purposes^(12)^.

Systems aimed at detecting falls aim to signal the occurrence of the event. Those intended for prediction and prevention work through wearable sensors, environmental sensors or software applications that collect and analyze data, sending the risk message to pre-determined equipment, speeding up health professionals’ work^(12,24-27)^. We observed a trend of inventions aimed at preventing falls, diverging from studies that pointed to detection as a priority, which may represent an important change in approach to patient safety^(12,24,28-29)^.

Wireless wearable devices stand out due to their ease of use and low cost, optimizing physical space and staff, in addition to adding multiple functionalities. Currently, it is possible to find equipment on the market, such as smart socks, which signal when leaving the bed, radiofrequency identification tags that can be attached to the chest and technology that can be attached to the upper part of the leg, all capable of identifying the movement of patients and forwarding risk information to a defined center^(24,30-32)^.

Among the technological strategies aimed at the environment, we have video monitoring, 3D image capture systems with a sensor capable of detecting movement, integrated alarm systems to signal the attempt and exit of patients from the bed and a wireless sensor placed on the bed with plural function used to prevent falls and pressure injuries, in addition to monitoring vital signs. Despite representing great progress, the adoption of imaging technologies faces obstacles due to patients’ lack of privacy and feelings of exposure. However, in some cases, acceptance is more positive than the presence of a caregiver^(27,33-36)^.

Given the wide range of devices available on the market, it is important to define the patient profile, risks and reported events as well as the type of fall and its causes so that the institution can define how to invest and which devices to implement.

Another trend in the technological resources investigated is the plurality of functions, acting, in addition to monitoring and preventing falls, in recording and analyzing vital parameters, which can assist in possible cases of clinical deterioration, in pressure injury prevention, among others. adverse events, which generate costs for the health system. The multiple functionality can, in addition to reducing the amount of equipment, optimize human resources and improve health team management^(27,31)^.

Technologies can provide greater agility and quality in care, bringing benefits to the care team and the patient, as it results in accuracy, constancy of actions and standardized storage of information, freeing nursing professionals from some technical activities, providing more time for planning and comprehensive coordination of care, ensuring safety of care, timely access, effectiveness of treatments and efficiency in the use of resources^(37)^.

Care team partnership is essential for the development and implementation of effective devices to prevent falls that are applicable in practice. In addition to acting as a parameter in the evaluation of new technologies and investigation of satisfaction and acceptance of products, nursing professionals should be encouraged to collaborate with ideas and viable alternatives to develop new products as well as register patents for their inventions^(38)^.

### Study limitations

It is worth highlighting the limitation of this prospection that it is not the objective of this work to finalize the study on patents aimed at preventing and signaling falls. It is worth highlighting that the topic deserves to be further explored in other databases and with the extension of the data collection period, as many patents may not be in the public domain due to the confidentiality protection period.

### Contributions to nursing, health, or public policies

Our study provides important information to nursing professionals about prevention technologies that can be used and adapted to different organizational, financial, social and cultural contexts, contributing to the search for quality and safety of care.

## CONCLUSIONS

The study revealed the predominance of developed nations, such as the United States, China, Japan and Korea, in the development of innovations for preventing and monitoring falls, considering international patent registration.

There is a trend towards wearable technological strategies aimed at prevention, even though many of the registered devices are intended for environments and signal the occurrence of a fall.

The presence of multiple functionalities in the same device highlights, in addition to the search for optimizing resources and better use of teams, the concern with comprehensive care.

Nursing professionals are responsible for collaborating with ideas and viable alternatives, developing new products and registering patents for their innovations.
